# Chatting While Walking Does Not Interfere with Topographical Working Memory

**DOI:** 10.3390/brainsci10110811

**Published:** 2020-11-02

**Authors:** Laura Piccardi, Alessia Bocchi, Massimiliano Palmiero, Maddalena Boccia, Simonetta D’Amico, Raffaella Nori

**Affiliations:** 1 Department of Psychology, “Sapienza” University of Rome, 00185 Rome, RM, Italy; alessia.bocchi@gmail.com (A.B.); maddalena.boccia@uniroma1.it (M.B.); 2 Cognitive and Motor Rehabilitation and Neuroimaging Unit, IRCCS Fondazione Santa Lucia, 00179 Roma, RM, Italy; 3 Department of Human and Social Sciences, University of Bergamo, 40126 Bologna, BO, Italy; massimiliano.palmiero@unibg.it; 4 Department of Biotechnological and Applied Clinical Sciences, University of L’Aquila, 67100 L’Aquila, Italy; simonetta.damico@univaq.it; 5 Department of Psychology, University of Bologna, 40127 Bologna, BO, Italy; raffaella.nori@unibo.it

**Keywords:** spatial, interference, navigation, orientation, working memory, frame of reference, gender

## Abstract

In the present study, we employed the dual task technique to explore the role of language in topographical working memory when landmarks are present along the path. We performed three experiments to mainly test the effects of language but also motor, spatial motor and spatial environment interferences on topographical working memory. We aimed to clarify both the role of language in navigational working memory per se and the extent to which spatial language interferes with the main task more than the other types of interference. Specifically, in the three experiments we investigated the differences due to different verbal interference sources (i.e., articulatory suppression of nonsense syllables; right and left, up and bottom; and north, south, east and west). The main hypothesis was that the use of spatial language affected more landmark-based topographical working memory than both the verbalization of nonsense syllables and other types of interference. Results show no effect of spatial language, only spatial environmental interference affected the navigational working memory performance. In general, this might depend on the scarce role of spatial language in online navigational working memory tasks. Specifically, language is more important for learning and retrieval of the cognitive map. Implications and future research directions are discussed.

## 1. Introduction

A landmark is a salient natural or artificial feature of the environment that stands out, working as a spatial reference [1,2,3]. The importance of having a view-dependent landmark representation to avoid getting lost is widely accepted. Undoubtedly, people take directional decisions during navigation based on landmark array representations [4]. MacDonald et al. [5] identified three different landmark-based strategies: (i) a *configural* strategy, by which the landmarks’ spatial array helps to identify the target (i.e., “I parked my car close to the postal office in front of the National Library”); (ii) a combination of *configural* and *elemental* strategies, which allows the processing of both distances and directions from the landmarks (i.e., “My car is at about 30 meters from the flower shop and on the left of the postal office”); (iii) the *beaconing* strategy, by which a landmark works as a beacon providing environmental suggestions to find the target (i.e., “I do not really remember where I parked my car, but it should be close to the National Library”).

Taking into account the key role of landmarks in everyday spatial orientation, it is not surprising that a critical step in children’s development is to learn to use single and multiple landmarks with the purpose of spatial orientation [6]. At the beginning, children are able to use single landmarks as direct markers of the goal location [7,8,9] and only later as indirect markers of the goal [6,10]. In general, children are unable to use multiple landmarks to find a hidden toy until they are in the preschool age (i.e., [11,12]). According to Siegel and White’s model [13], the spatial knowledge evolves from landmark (i.e., such as a figurative memory of environmental objects) to route (i.e., an egocentric perspective in which the relation between landmarks met along the path is coded through the own body) and then from route to survey (i.e., an allocentric perspective that implies a map-like representation) knowledge. At each stage, new skills are acquired. However, there is an ongoing debate about the assumptions of this seminal model, especially concerning its cumulative and hierarchical structure as well as the ancillary almost non-existent role of spatial language. Therefore, while still a key theoretical model in the literature on spatial orientation, other models have proposed different ways of acquiring environmental information, as well as different steps for the acquisition. For example, Tversky [14] highlights the importance of language for human beings with respect to other species very skilled in navigation. Tversky hypothesized the existence of a further stage, only for humans, in which environmental representations are integrated with linguistic spatial categories. Montello [15], on the other hand, proposed the “Continuous Model”, which supports the idea of a continuous metric development and allows the acquisition of survey knowledge even after a few seconds of environmental exposure.

Undoubtedly, spatial language and spatial orientation are closely linked to each other in the early years of development. Piccardi et al. [16] found that the more proficient children are in understanding spatial locatives, the more they are able to learn pathways, retrieve them after a delay and represent them on a map when landmarks are present in the environment. These findings suggest that comprehension of linguistic spatial categories has a key role when individuals rely on sequences of landmarks to drive their navigation towards a given goal. Nevertheless, the role of language seems to be less important when landmarks are not present and individuals have to use the geometric shape of the environment or when paths to remember are short and coding the own body movements is enough to reach the target or to come back (e.g., [17,18]).

Based on the idea that a task can interfere with another task only if it uses the same cognitive resources, dual-task paradigms allow for testing mechanisms underlying specific spatial components. Indeed, through the specific interference paradigm it is possible to test the existence of different components within the visuo-spatial working memory (VSWM, e.g., [19,20]). When the main task is associated with a secondary interfering task, a suppression effect occurs. For example, secondary tasks that interfere with the VSWM are spatial tapping, pointing, voluntary eye movements, attentional shift, movements of legs and arms. All of these tasks may reduce the participants’ performance of VSWM. In a previous study, Piccardi and co-workers [17], investigating the nature of topographical working memory, found that only one component affected the performance, namely spatial environmental interference. Specifically, they asked participants to repeat short paths on a large space after having observed the examiner perform them. While participants were observing the examiner, different interference tasks were administered. Specifically, in the articulatory suppression, the participants had to repeat without meaning syllables. In the motor interference, the participants were asked to walk on the spot, whereas during the spatial motor interference, the participants were required to bend his/her leg at knee level and then stretch it out backward, alternating the left and right leg, always standing in the same place while the investigator was illustrating the sequence. Finally, in the spatial environmental interference, participants were asked to point with their index finger to the source of a sound coming from a PC every 2 s from four random different positions (i.e., in front, behind, on the right, or on the left) while the investigator was illustrating the sequence. Findings show that topographical working memory was only impaired when participants pointed at different sound positions in the environment; in other words, only the environmental interference reduced the performance of topographical working memory. However, landmarks were not displayed, and this may explain the reason why the verbal interference did not affect the participants’ performance.

In the present study, we aimed at investigating whether the presence of landmarks in the navigational vista space (the space that can be visually apprehended from a single location or with only little exploratory movements, Wolbers and Wiener [21]) requires different information processing including some linguistic aspects inherent to spatial language as indicators of direction and position. Specifically, we hypothesized that when landmarks were available in the environment, participants could also use language to anchor their position in the array and with respect to themselves. To this purpose, we performed three different experiments in which the verbal interference was changed. In the first one, participants performed a classical articulatory suppression task in which the examiner asked them to repeat syllables without meaning. In the second experiment, instead the examiner asked participants to repeat words with underlying egocentric representation, such as right, left, back and forth and in the last experiment, the examiner asked participants to repeat words with underlying allocentric representation, that is to say, north, south, east and west.

It is known, indeed, that right, left, back and forth are egocentric terminologies, while the cardinal points are considered allocentric terminologies that can convey either a route or a survey perspective to an individual (e.g., [22,23]). For this reason, we do not expect to observe an effect in the first experiment using a classical articulatory suppression task (a generic verbal interference), but we expect to observe an effect in the second experiment using the terms underlying the egocentric representation, as in the working memory topographical task the participant maintains a first-person perspective (egocentric) while observing the examiner illustrating the path. For such a reason, we hypothesized that participants would show a poorer performance when the interfering task requires using the same egocentric terms used to store the path previously illustrated by the examiner. On the contrary, we do not expect effects with allocentric terms that do not correspond to the perspective that the subject takes during the observation of the paths made by the examiner. Furthermore, the allocentric terms require a cognitive transformation from an egocentric to a more abstract allocentric representation. Furthermore, spatial language when associated with landmarks is more often associated with egocentric terms than with allocentric ones. For example, when an individual provides directional information, they will tend to explain the route by using more egocentric terms than allocentric terms, even if this tendency could be related to individual navigational strategies.

## 2. Materials and Methods

### 2.1. Overview of the Experiments

#### 2.1.1. Ethic Statement

The three experiments described hereafter met the criteria of the Declaration of Helsinki and were approved by the Institutional Ethical Board. Specifically, the Research Ethics Committee of the Psychology Department of Bologna University approved the study (prot. n. 118,423) on 30 May 2019.

All participants signed an informed consent form before starting the experiment that they took part in.

#### 2.1.2. Participants

In all three experiments, no participant has a history of psychiatric or neurological disorders or spatial orientation disorders, and no participant reports the presence of developmental topographical disorientation (as evaluated by the Familiarity and Spatial Cognitive Style Scale: [24]), a neurodevelopment disorder characterized by a specific and selective deficit in environmental learning that affects healthy individuals (e.g., [25,26]). To exclude general visuo-spatial and topographical short-term memory deficits, all subjects were administered the Corsi Block-Tapping Test (CBT; [27,28]) and the Walking Corsi Test (WalCT: [28,29]). All participants performed the CBT and WalCT well above the cut-off for their age, gender and schooling (in accordance with normative data reported in [28]). Failure in these two tests was considered an exclusion criterion. Demographic details of participants who took part in the three experiments are reported below in the specific sections.

#### 2.1.3. Apparatus

**Figure 1:**
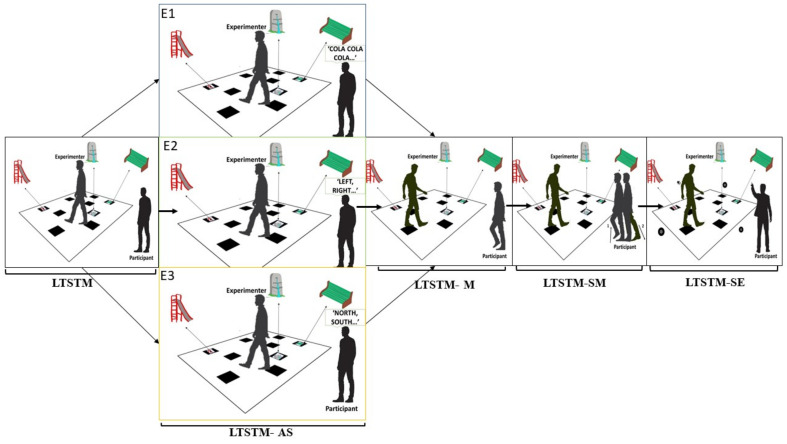
Experimental design and procedure. From left to right, figures show: the Walking Corsi Test (WalCT) administered to assess landmark topographical short-term memory (LTSTM), and the 4 dual-task interference conditions (articulatory suppression: LTSTM-AS; motor interference: LTSTM-M; spatial motor interference: LTSTM-SM and spatial environmental interference: LTSTM-SE). In the LTSTM-AS, participants were asked to repeat an irrelevant speech sound (i.e., COLA-COLA-COLA-COLA) (first experiment: E1), four egocentric spatial terms (i.e., right, left, back and forth; right, left, back and forth…) (second experiment: E2), or four allocentric spatial terms (i.e., north, south, east, west; north, south, east, west) (third experiment: E3) when the examiner was showing the sequence and when they were repeating the sequence just seen. In the LTSTM-M condition, participants walked on the spot while the examiner was showing the sequence and then they had to repeat it. In the LTSTM-SM condition, participants repeatedly made a sequence of leg movements, that is, they had to bend their leg at knee level and then stretch it out backward alternating the left and right leg, always standing in the same place, while the examiner was showing the sequence. Afterwards, they repeated the just seen sequence. In the LTSTM-SE condition, participants were asked to point with their index finger to the source of a sound coming from a PC every 2 s from four random different positions (in front, back, on the right or on the left) while the examiner was showing the sequence, then they had to repeat the sequence previously observed.

The Walking Corsi Test (WalCT: [28,29] see Figure 1) was used for the three experiments. WalCT allows for testing topographical memory in a vista-navigational space (i.e., a space corresponding to the portion of navigational space that can be experienced from a single location or with only little exploratory movements; [21]). It consists of nine squares (30 × 30 cm) placed on the floor (3 × 2.5 m) and it represents a large-scale version of the well-known Corsi Block-Tapping Test (CBT: [27,28]). The squares are placed on the floor respecting the same position of the cubes in the CBT. The room in which WalCT is placed is empty. Different span sequences, balanced for degree of difficulty, as tested in Piccardi et al. [17], were used in the 4 different dual-task interference conditions. Three 30 × 30 cm pictures of landmarks (a fountain, a bench, a slide, see Figure 1) were placed on three out of the nine WalCT squares, the landmarks’ position varied across sequences and landmarks were placed in the main turning points of the short paths. The landmarks were clearly visible to the participants during all the experiments, both while observing the experimenter showing the different paths, and when they had to repeat the path previously seen.

During the WalCT, the examiner illustrated the sequence by walking across the WalCT test [28,29] and stopping on each square for 2 s. The participant then had to repeat the same sequence as the examiner by walking and stopping on the same previously shown squares. The sequences gradually increased in length (starting from a two-square sequence up to a nine-square sequence), and the score was calculated on the number of squares in the longest sequence remembered correctly (square span).

#### 2.1.4. Procedure

The WalCT was administered to assess landmark topographical short-term memory (LTSTM) in 4 dual-task interference conditions (articulatory suppression (AS); motor interference (M); spatial motor interference (SM) and spatial environmental interference (SE)). In the AS condition, participants were asked to repeat an irrelevant speech sound (i.e., COLA-COLA-COLA-COLA) (first experiment), four egocentric spatial terms (i.e., right, left, back and forth; right, left, back and forth…) (second experiment), or four allocentric spatial terms (i.e., north, south, east, west; north, south, east, west) (third experiment) when the examiner was showing the sequence and when they were repeating the sequence just seen (see Figure 1). In the M condition, participants walked on the spot while the examiner was showing the sequence and then they had to repeat it (see Figure 1). In the SM condition, participants repeatedly made a sequence of leg movements, that is, they had to bend their leg at knee level and then stretch it out backward alternating the left and right leg, always standing in the same place, while the examiner was showing the sequence. Afterwards, they repeated the just seen sequence (see Figure 1). In the SE condition, participants were asked to point with their index finger to the source of a sound coming from a PC every 2 s from four random different positions (in front, back, on the right or on the left) while the examiner was showing the sequence, then they had to repeat the sequence previously observed. This procedure was the same as described by Wen et al. [30] and then adopted by Piccardi et al. [17] (see Figure 1). The administration order of the four dual-task interferences was counterbalanced across subjects to avoid facilitation effects on one interference condition with respect to the others.

#### 2.1.5. Statistical Analysis

A 2 × 5 mixed factorial ANOVA was performed for each of the three experiments, with gender as a between factor and task as a repeated measure (LTSTM, LTSTM + M, LTSTM + SM, LTSTM + AS, LTSTM + SE). Gender differences were also checked because in previous studies men showed better short-term topographical memory than women [17,28,29]. The Bonferroni’s correction for multiple comparisons was used as post-hoc comparisons.

### 2.2. Differences in Each Experiment 

#### 2.2.1. Experiment 1: Articulatory Suppression with Syllables without Meaning

##### Participants

Forty students from the Department of Biotechnological and Applied Clinical Science, University of L’Aquila (L’Aquila, Italy) participated (20 females; mean age = 23.5; standard deviation = 2.82; age range = 19–30 years). Only right-handed subjects were included [31].

#### 2.2.2. Experiment 2: Articulatory Suppression with Words Underlying Egocentric Representation

##### Participants

Forty students were also recruited from the Department of Biotechnological and Applied Clinical Science, University of L’Aquila (L’Aquila, Italy) (24 females; mean age = 24.6, standard deviation = 3.27; age range = 20–36 years). Only right-handed subjects were recruited [31].

##### Procedure

Participants performed a one-task condition and the four dual-task conditions (AS, M, SM and SE) with landmarks. Only the AS condition was administered in a different way, as follows: participants were requested to continuously repeat four egocentric spatial terms (i.e., right, left, back and forth; right, left, back and forth…) while the examiner was illustrating the sequence and while repeating the sequence just seen (see Figure 1). The order of presentation of the conditions was randomized among participants.

#### 2.2.3. Experiment 3: Articulatory Suppression with Words Underlying Allocentric Representation

##### Participants

Forty students of the Department of Biotechnological and Applied Clinical Science, University of L’Aquila (22 females; mean age = 20.38, standard deviation = 1.44; age range = 19–24 years) volunteered to participate in experiment three. All participants were right-handed [31].

##### Procedure

Additionally, in experiment three, participants performed a one-task condition and the four dual-task conditions (AS, M, SM and SE) with landmarks. M, SM and SE were performed as in the other experiments, but the AS condition was administered as follows: participants were requested to continuously repeat four egocentric spatial terms (i.e., north, south, east and west; north, south, east and west …) while the examiner was illustrating the sequence and while repeating the sequence just seen (see Figure 1). The order of presentation of the conditions was randomized among participants.

## 3. Results

### 3.1. Experiment 1: Articulatory Suppression with Syllables without Meaning

None of the participants was impaired in visuo-spatial working memory (VSWM; mean = 6.08, SD = 0.94; cut-off < 3.69 in [28]) or topographic short-term memory (TSTM; mean = 5.18, SD = 0.78; cut-off < 3.14, as in [28]).

**Figure 2:**
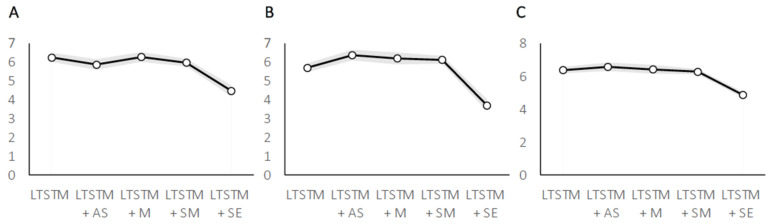
Lines depict average performances in experiment 1 (**A**), experiment 2 (**B**) and experiment 3 (**C**). Shadow lines depict standard error.

The ANOVA showed a main effect of gender (F (1, 38) = 4.6658, *p* = 0.037; partial η^2^ = 0.109), men (mean = 6.17, SE = 0.26) performed better than women (mean = 5.37, SE = 0.26). In addition, the analysis showed a main effect of task (F (4, 152) = 15.015, *p* = 0.000001; partial η^2^ = 0.283), post-hoc comparisons (Bonferroni: *p* < 0.00001) revealed that the LTSTM + SE dual-task condition (mean = 4.48; SE = 0.29) produced lower scores than LTSTM (mean = 6.25; SE = 0.25), LTSTM + M (mean = 6.28; SE = 0.26), LTSTM + SM (mean = 5.98; SE = 0.21 ) and LTSTM + AS (mean = 5.88; SE = 0.28) dual-task conditions. No further significant differences were found between conditions. The interaction effect of gender and task was not significant (F (4, 152) = 1.0345, *p* = 0.39; partial η^2^ = 0.027). Table 1 shows means, standard deviations and standard errors (see Figure 2).

### 3.2. Experiment 2: Articulatory Suppression with Words Underlying Egocentric Representation

None of the participants was impaired in VSTM (mean = 5.95, SD = 1.13; cut-off < 3.69 in [28]) or TSTM (mean = 5.3, SD = 0.94; cut-off < 3.14, as in [28]).

The ANOVA showed a main effect of gender (F (1, 38) = 15.646, *p* = 0.0003; partial η^2^ = 0.292), men (mean = 6.44, SE = 0.27) were better than women (mean = 5.08, SE = 0.22). In addition, the analysis also showed a main effect of task (F (4, 152) = 29.472, *p* = 0.0000001; partial η^2^ = 0.437), post-hoc comparisons (Bonferroni: *p* < 0.0000001) revealed that the LTSTM + SE dual-task condition (mean = 3.74; SE = 0.33) produced lower scores than LTSTM (mean = 5.86; SE = 0.21), LTSTM + M (mean = 6.44; SE = 0.27), LTSTM + SM (mean = 6.21; SE = 0.20) and LTSTM + AS (mean = 6.53; SE = 0.26) dual-task conditions. No further significant differences were found between conditions. The interaction effect of gender and task was also significant (F (4, 152) = 3.251, *p* = 0.014; partial η^2^ = 0.079), post-hoc comparisons (Bonferroni: *p* < 0.05) revealed that men (mean = 7.63; SE = 0.41) were better than women (mean = 5.25; SE = 0.34) only in the LTSTM + M. In general, both males and females produced lower scores in the LTSTM + SE dual-task condition as compared to other conditions. Table 2 shows means, standard deviations and standard errors.

### 3.3. Experiment 3: Articulatory Suppression with Words Underlying Allocentric Representation

None of the participants was impaired in VSTM (mean = 6.15, SD = 1; cut-off < 3.69 in [28]) or TSTM (mean = 5.15, SD = 0.83; cut-off < 3.14, as in [28]).

The ANOVA showed a main effect of gender (F (1, 38) = 6.201, *p* = 0.017; partial η^2^ = 0.140), men (mean = 6.5, SE = 0.21) were better than women (mean = 5.78, SE = 0.19). In addition, the analysis showed a main effect of task (F (4, 152)= 16.674, *p* = 0.000001; partial η^2^ = 0.305), post-hoc comparisons (Bonferroni: *p* < 0.0000001) revealed that the LTSTM + SE dual-task condition (mean = 4.91; SE = 0.18) produced lower scores than LTSTM (mean = 6.38; SE = 0.20), LTSTM + M (mean = 6.47; SE = 0.26), LTSTM + SM (mean = 6.31; SE = 0.16) and LTSTM + AS (mean = 6.63; SE = 0.25) dual-task conditions. No further significant differences were found between conditions. The interaction effect of gender and task was not significant (F (4, 152) = 1.315, *p* = 0.27; partial η^2^ = 0.033). Table 3 and Figure 2 show means, standard deviations and standard errors.

## 4. Discussion

### 4.1. Experiment Parts

#### 4.1.1. Experiment 1: Articulatory Suppression with Syllables without Meaning

The aim of the first experiment was to investigate what kind of interfering task would reduce the topographical short-term memory performance in the presence of landmarks. Assuming that spatial language plays a role in relating the position of the individual landmarks to each other and their sequence within the path, as well as the position of the individual along the path and with respect to the landmarks, it is possible that even a simple articulatory suppression task could worsen the performance. Concerning gender differences, our results confirm that males performed better in all conditions, both in one-task and dual-task conditions. Moreover, with respect to the main aim, these results extend Piccardi et al.’s [17] study, since it emerged that even with the presence of landmarks, the only interfering condition that affected the topographical performance was the SE condition, in which participants had a smaller span. The articulatory suppression did not compromise the execution of the task.

#### 4.1.2. Experiment 2: Articulatory Suppression with Words Underlying Egocentric Representation

The present topographical memory task requires an egocentric perspective, because the participant observed the examiner showing the sequence and then repeated it. For this reason, the hypothesis was that with the presence of landmarks not only the environmental interference would reduce the performance, but also the articulatory suppression in which the subjects repeated right, left, back and forth. Results confirm once again the presence of gender differences, men were better than women, specifically in the dual-task condition with motor interference. Both genders performed worse in the SE condition. In addition, the only interference condition that significantly reduced the performance was the SE condition, no significant effects emerged in the AS condition.

#### 4.1.3. Experiment 3: Articulatory Suppression with Words Underlying Allocentric Representation

In the present experiment, we assessed whether the landmark topographical working memory could be reduced when participants performed an interfering task using allocentric terms, such as cardinal points, hypothesizing that even if the learning perspective was in first-person when subjects repeat the just observed sequence, they could handle a mental representation of it in the WalCT (similar to a map). Results show that men outperformed women and that the only interference condition that reduced performance was the environmental condition (SE), no significant effects emerged in the AS condition.

### 4.2. General Discussion for all Experiments

The space around us is represented into different frames of reference, namely body-centered and world-centered. The interaction between these two representations allows placing landmarks and their spatial location in respect to us and to other environmental objects. Whenever landmarks are present in an environment, the individual tends to associate verbal labels that link the landmark to the next one with the direction to take. For this reason, we have hypothesized that the landmark-based navigational working memory could be affected by verbal interference. In a previous study, Piccardi et al. [17] found that only environmental interference, in which the subjects had to indicate the origin of a sound in the environment, interfered with the navigational working memory. However, in this experiment, no landmark was present in the environment. In the present study, through a series of experiments, we investigated the effect of verbal interference using different types of verbal material, meaningless syllables, spatial egocentric terms and spatial allocentric terms. Our hypothesis was that in a task where the participant’s perspective was in first person, the egocentric terms would interfere more at the time of acquisition. Moreover, in the general population, differences in using navigational strategies have been described and three different navigational strategy (landmark, route, and survey) users have been identified. Considering that most of the population is route user, this has allowed us to assume that mainly egocentric terms could reduce the performance. We also consider spatial allocentric terms because part of the participants would solve the WalCT in an allocentric way, as they view the route from the side of the environment. However, results did not confirm the initial hypothesis, as well as an interference due to spatial allocentric terms.

The three experiments consistently show that only environmental interference significantly reduced participant’s performance. In addition, across experiments, men showed better navigational working memory than women regardless of the type of interference.

How can we interpret these results? It is possible that language, although it has an undisputed role within some navigational models (e.g., [14]) and has a fundamental role for us especially in providing directional instructions, communicating our position and sharing environmental knowledge with our co-specifics, does not play a role in keeping environmental information online. It is also possible that language, particularly spatial language, may play a role at specific moments in the life span or in the presence of neurological pathologies and/or neurodevelopment diseases. In fact, Piccardi et al. [16] showed how children’s competence of understanding space locations predicted their ability to learn paths. It is also possible to imagine that this aspect may be present in the elderly, navigational working memory deficits already being present in the early stages of Alzheimer’s disease [32]. Another possibility is that language makes a contribution at different times in the navigation process, for example, during the learning and retrieval of the cognitive environmental map, and that its weight is less in keeping temporary information online, as was the case in our experiments, which evaluated verbal interference during navigational working memory tasks. To give a real-life example, we used this skill while finding our way back out of a shopping center that we had never entered before. During navigation, we used every day different temporal aspects of navigational memory and this determined a successful spatial orientation process.

Although the presence of gender differences was a secondary purpose, as we expected, men outperformed women (e.g., [33,34,35,36,37]). We also expected gender difference to be more pronounced in verbal interference because previous studies have reported that women tend to use verbal strategies more than men during navigation. However, we observed that men were better than women despite the type of interference and also, for them, environmental spatial interference made the task more difficult.

Taken together, these results contribute to clarify what type of interference may hamper performances during landmark-based navigational working memory tasks. When landmarks are present, the spatial-environmental interference is the only one that affects topographical working memory. In these experiments (similar to in [17,18]), motor and spatial-motor interferences do not impair performance on the landmark WalCT, supporting the evidence that the motor component is automatic and not explicitly processed during navigation. In line with this, patients with cerebellar deficits also showed no problem with the WalCT due to motor impairment [38]. These findings also shed some light on the role of spatial language in landmark-based working memory, suggesting that even in this case language represents something that we usually use as we move around the environment without being affected by it. For example, it is customary to take a walk with friends and talk amiably, but this does not prevent us from retracing our steps or retracing our steps in reverse. Thus, it is possible, as we mentioned above, that language has a fundamental role in building, representing, and recollecting the sequences of the environmental cognitive map and not in the transient holding and processing of new environmental information.

Undoubtedly, future studies should take into account the different navigational strategies, stratifying the sample on the basis of navigational styles to really understand whether individuals with route style suffer more from verbal interference with egocentric terms when compared to landmark and survey users or whether, in spite of the navigational strategy, the spatial language does not affect topographical working memory.

Furthermore, future studies should investigate the effect of verbal interference in pathological populations. For example, in patients with mild cognitive impairment, where recently navigational memory appears to be one of the neuropsychological markers for the earlier identification of the disorder [39], or in the early stages of Alzheimer’s disease, or in brain-damaged patients with aphasia, or even in healthy subjects with developmental topographical disorientation, navigational memory seems to be an element that can allow the creation of a specific taxonomy of the disorder to emphasize the agnostic aspects against the amnestic ones (see, for example, [40]). Surely investigating the effects of interferences on neurological pathologies will help both the diagnosis and the planning of targeted rehabilitation interventions.

## 5. Conclusions

In conclusion, the present series of experiments show that verbal interference does not affect the topographic working memory even when landmarks are present. This type of interference does not occur even when verbal interference requires the participant to produce egocentric and allocentric spatial terms. Interestingly the only interference able to reduce performance is those environmental when participants have to point a sound locates in the room. Undoubtedly, our data throws some light on the topographic working memory and its characteristics.

## Figures and Tables

**Table 1:** Descriptive statistics: means (standard deviations) and standard errors (SE).

	LTSTM	LTSTM + M	LTSTM + SM	LTSTM + AS	LTSTM + SE
Females	5.65 (1.53) SE = 0.34	5.95 (1.79) SE = 0.40	5.6 (1.10) SE = 0.25	5.75 (1.65) SE = 0.37	3.9 (1.37) SE = 0.31
Males	6.85 (1.39) SE = 0.31	6.6 (1.47) SE = 0.33	6.35 (1.42) SE = 0.32	6 (1.95) SE = 0.44	5.05 (2.06) SE = 0.46
Total	6.25 (1.57) SE = 0.25	6.28 (1.65) SE = 0.26	5.98 (1.31) SE = 0.21	5.88 (1.79) SE = 0.28	4.48 (1.83) SE = 0.29

Note: LTSTM = landmark topographic short-term memory; + M = motor; + SM = spatial-motor; + AS; articulatory suppression; + SE = spatial environmental.

**Table 2:** Descriptive statistics: means (standard deviations) and standard errors (SE).

Groups	LTSTM	LTSTM + M	LTSTM + SM	LTSTM + AS	LTSTM + SE
Females	5.04 (1.49) SE = 0.30	5.25 (1.73) SE = 0.35	5.79 (1.35) SE = 0.28	5.75 (1.7) SE = 0.35	3.54 (1.64) SE = 0.34
Males	6.69 (0.87) SE = 0.22	7.63 (1.54) SE = 0.39	6.63 (1.02) SE = 0.26	7.31 (1.49) SE = 0.37	3.94 (2.52) SE = 0.63
Total	5.86 (1.51) SE = 0.21	6.44 (2.02) SE = 0.27	6.21 (1.28) SE = 0.20	6.53 (1.78) SE = 0.26	3.74 (2.05) SE = 0.33

Note: LTSTM = landmark topographic short-term memory; + M = motor; + SM = spatial-motor; + AS; articulatory suppression; + SE = spatial environmental.

**Table 3:** Descriptive statistics: means (standard deviations) and standard errors (SE).

	TSTM-L	TSTM-L + M	TSTM-L + SM	TSTM-L + AS	TSTM-L + SE
Females	6.32 (1.25) SE = 0.27	6 (1.51) SE = 0.32	5.95 (0.9) SE = 0.19	6.05 (1.84) SE = 0.39	4.59 (1.14) SE = 0.24
Males	6.44 (1.29) SE = 0.30	6.94 (1.7) SE = 0.4	6.67 (1.08) SE = 0.26	7.22 (1.11) SE = 0.26	5.22 (1.06) SE = 0.25
Total	6.38 (1.25) SE = 0.20	6.47 (1.65) SE = 0.26	6.31 (1.04) SE = 0.16	6.63 (1.65) SE = 0.25	4.91(1.14) SE = 0.18

Note: TSTM = topographic short-term memory; -L = landmark; + M = motor; + SM = spatial-motor; + AS; articulatory suppression; + SE = spatial environmental.
